# An unusual cause of status epilepticus

**DOI:** 10.4103/0972-5229.56060

**Published:** 2009

**Authors:** Supradip Ghosh, Alok Ahlawat, Krishna Kumar Rai, Ashu Arora

**Affiliations:** **From:** (Internal Medicine) Consultant, Critical Care and In Charge ICU Sarvodaya Hospital and Research Centre, YMCA Road Sector – 8, Faridabad, Haryana, India; 1 ICU Sarvodaya Hospital and Research Centre, YMCA Road Sector – 8, Faridabad, Haryana, India; 2 Consultant Physician Arvodaya Hospital and Research Centre, YMCA Road Sector – 8, Faridabad, Haryana, India

**Keywords:** Deltamethrin, pyrethroid insecticide, status epilepticus

## Abstract

A 24-year-old female presented with status epilepticus following ingestion of a pyrethroid insecticide Deltamethrin. The pathophysiology, clinical features, and management of pyrethroid poisoning are discussed in this article.

## Introduction

Pyrethrins are natural extracts derived from flowers of chrysanthemam cinerarifolium and C. cocineum. Pyrethroids are synthetic analogues of these natural abstracts. Pyrethroids are widely used as insecticides and are also used in the topical treatment of scabies and lice. Despite being used extensively, there are only a few reports of human pyrethroid poisoning. Because of their increased sodium channel sensitivity, smaller body size, and lower body temperature, insects are 2,250 times more prone to toxicity by pyrethroids than humans.[1] In addition, humans are relatively protected from pyrethroids because of their poor dermal absorption and rapid metabolism to non toxic metabolites. Fewer then 10 deaths have been reported from the ingestion of pyrethroids or from occupational exposure.[1] We describe a case of Deltamethrin poisoning presenting with status epilepticus.

## Case Report

A 24-year-old female was admitted to our intensive care unit (ICU) with repeated episodes of seizures following deliberate ingestion of an unknown quantity of the anti-lice medication BUTOX (Deltmethrin 1.2%). On arrival in the emergency department, the patient was given a bolus dose of Lorazepam, and soon intubated, and then a loading dose of phenytoin was commenced. Gastric lavage was not performed as patient had repeated convulsions. On examination, she was deeply comatose, and was getting generalized tonic clonic seizures. Her pulse rate was 130/min regular, and her blood pressure was 136/76 mmHg. Her pupils were equal (2 mm in size) bilaterally briskly reacting to light. Other systemic examinations were unremarkable. An arterial blood gas showed mild metabolic acidosis. Hemogram, renal and liver function tests, electrocardiogram, and chest X-ray were normal. After repeat doses of Lorazepam and phenytoin, the patient was put on mechanical ventilatory support. A therapeutic coma was induced using a combination of Midazolam and Thiopentone sodium, as the patient continued to have convulsions. In the next 36 hours, Thiopentone and Midazolam were gradually tapered off after electroencephalography documentation of complete suppression of burst activity. She was gradually weaned off the ventilator after 72 hours. She was discharged from the hospital on Day 5 after psychiatry consultation.

## Discussion

On the basis of their chemical structure, pyrethroids are divided into two groups: Type I pyrethroids are devoid of a cyano moiety at the alpha-position of the basic cyclopropane carboxylic ester structure (e.g., Allethrin) while Type II pyrethroids have an alpha-cyano moiety (i.e., Fenvalerate and Deltamethrin). Pyrethroids produce prolonged opening of membrane sodium channels resulting in membrane depolarization, repetitive discharges, and synaptic disturbances leading to hyperexcitatory symptoms of poisoning.[3] Only low pyrethroid concentrations are necessary to modify sensory neurone function. Type II pyrethroids also decrease chloride currents through voltage-dependent chloride channels and this action probably contributes the most to the features of poisoning with Type II pyrethroids.[1] At relatively high concentrations, pyrethroids can also act on gamma-aminobutyric acid-gated chloride channels, which may be responsible for the seizures seen with severe Type II poisoning.[1] There are suggestions that voltage-sensitive calcium (Ca(2+)) channels (VSCC) may also be important targets of pyrethroid action. However, currently the data available neither supports nor refutes conclusively the hypothesis that effects on VSCC are important to the acute neurotoxicity of pyrethroids.[4]

Type I pyrethroids cause a Type I poisoning syndrome characterized by reflex hyperexcitability and fine tremor, whereas Type II pyrethroids produce salivation, hyperexcitability, choreoathetosis, and seizures.[1,2] Both produce potent sympathetic activation. Local contamination of the skin produces paresthesias - the face being most commonly affected. The paresthesiae are exacerbated by sensory stimulation such as heat, sunlight, scratching, sweating, or the application of water. Ingestion produces gastric irritation. The possibility that they also induce hypersensitivity reaction, which may be fatal when the respiratory tract is involved, has been debated for many years.[5]

Because there is no antidote for pyrethrin and pyrethroid poisoning, treatment is symptomatic and supportive. Pyrethroid paresthesias are treated by decontamination of the skin. Seizures due to systemic poisoning is sometimes difficult to control with anticonvulsants.[2] Pentobarbitone, however, is surprisingly effective as therapy against systemic Type II pyrethroid poisoning in rats, probably due to its dual action as a chloride channel agonist and a membrane stabilizer.[2]

Seizures are a known manifestation of pyrethroid poisoning. In a series of five cases described from India, one patient had convulsions.[6] Two children presented with generalized tonic-clonic convulsions following Allethrin ingestion.[7] However, after an extensive Internet search, we could not find a single case report of pyrethroid poisoning presenting with status epilepticus.
